# Supplementary material for: Estrogen Modulates Epithelial Breast Cancer Cell Mechanics and Cell-to-Cell Contacts
Source: Materials (Basel). 2021 May 28;14(11):2897. doi: 10.3390/ma14112897 (PMC8198807; doi:10.3390/ma14112897)
Supplement: Supplementary file 1 [file materials-14-02897-s001.zip › materials-1233074-supplementary.pdf]

materials

Supplementary Materials

Estrogen Modulates Epithelial Breast Cancer Cell Mechanics and Cell-to-Cell Contacts

Barbara Zbiral [1,†], Andreas Weber [1,*,†], Jagoba Iturri [1], Maria d. M. Vivanco [2] and José L. Toca-Herrera [1,*]

[1] Institute for Biophysics, Department of Nanobiotechnology, University of Natural Resources and Life Sciences, Muthgasse 11, 1190 Vienna, Austria; barbara.zbiral@boku.ac.at (B.Z.); Jagoba.iturri@boku.ac.at (J.I.)
[2] CIC bioGUNE, Basque Research and Technology Alliance, BRTA, Bizkaia Technology Park, 48160 Derio, Spain; mdmvivanco@cicbiogune.es
* Correspondence: andreas.weber@boku.ac.at (A.W.); jose.toca-herrera@boku.ac.at (J.L.T.-H.)
† These authors have contributed equally.

Citation: Zbiral, B.; Weber, A.; Iturri, J.; Vivanco, M.d.M.; Toca-Herrera, J.L. Estrogen Modulates Epithelial Breast Cancer Cell Mechanics and Cell-to-Cell Contacts. *Materials* **2021**, *14*, 2897. https://doi.org/10.3390/ma14112897

Academic Editor: Claudio Canale and Ornella Cavalleri

Received: 7 May 2021
Accepted: 25 May 2021
Published: 28 May 2021

Publisher's Note: MDPI stays neutral with regard to jurisdictional claims in published maps and institutional affiliations.

Copyright: © 2021 by the authors. Licensee MDPI, Basel, Switzerland. This article is an open access article distributed under the terms and conditions of the Creative Commons Attribution (CC BY) license (http://creativecommons.org/licenses/by/4.0/).

1. Short-term treatment with Estrogen

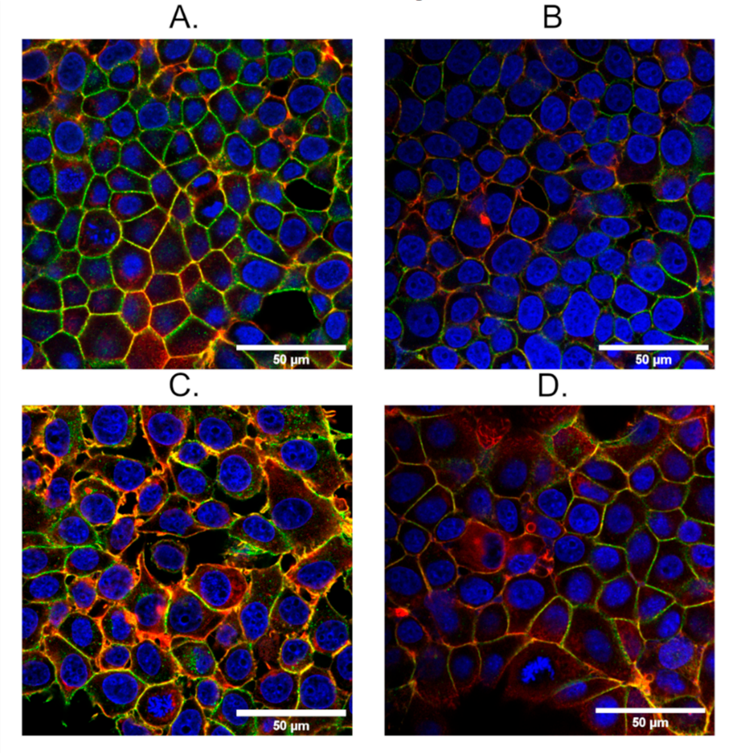

Figure S1. Short term (6 hour) treatment of MCF-7 cells with Estrogen (A–CTL DMEM, B–DMEM E_2, C–CTL HF, D–HF E_2). No significant changes due to estrogen treatment can be seen.

2. Performance of fitting stress relaxation data

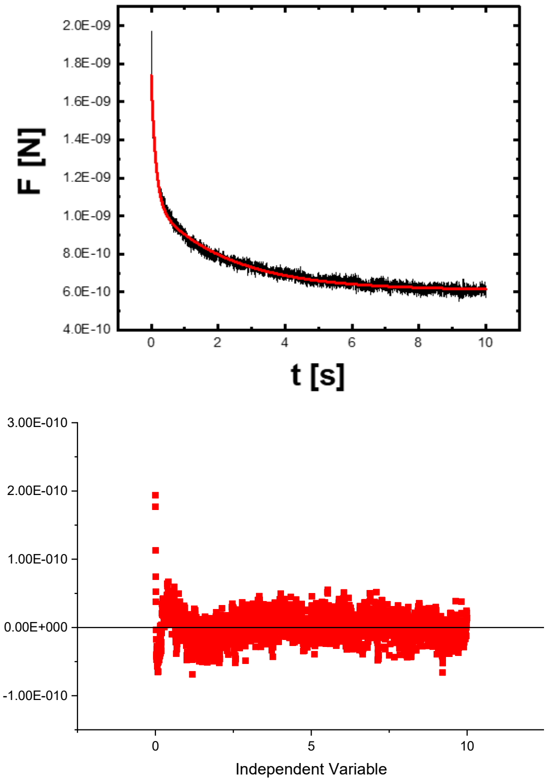

Figure S2. Fitting of the used viscoelastic model. (Left) Fitting of the stress relaxation curve using the model as described in Materials and Methods section. (Right) Residuals showing the very good agreement of the fitting with the data.

3. Statistical Properties and Analysis of Viscoelastic Properties

3.1. Measurements performed with tips

3.1.1. Elastic properties

Table S1. Apparent Young's Modulus values (in kPa) for MCF-7 cells measured with tips, shown are the number of measurements, the mean value, the standard deviation, the standard error of the mean value and the median.

	N	Mean	SD	SE	Median
CTL DMEM	371	3.11	1.41	0.07	3.06
DMEM E_2	387	2.59	1.13	0.06	2.41
CTL HF	343	3.10	1.27	0.07	2.93
HF E_2	345	2.18	0.92	0.05	2.05

Table S2. Statistical analysis of apparent Young's Modulus values for MCF-7 cells (one-way ANOVA with post-hoc Tukey test). Differences with statistical significance are shown as bold.

	CTL DMEM	DMEM E_2	CTL HF	HF E_2
CTL DMEM		**1.47E-8**	0.99	**1E-20**
DMEM E_2	**1.47E-8**		**5.92E-8**	**3.01E-5**
CTL HF	0.99	**5.92E-8**		**1E-20**
HF E_2	**1E-20**	**3.01E-5**	**1E-20**	

Table S3. Indentation values (in μm) for MCF-7 cells measured with tips, shown are the number of measurements, the mean value, the standard deviation, the standard error of the mean value and the median.

	N	Mean	SD	SE	Median

CTL DMEM	371	2.06	0.50	0.01	2.05
DMEM E_2	387	2.38	0.64	0.01	2.35
CTL HF	343	2.04	0.49	0.01	2.02
HF E_2	345	2.62	0.61	0.01	2.53

Table S4. Statistical analysis of apparent Young's Modulus values for MCF-7 cells (one-way ANOVA with post-hoc Tukey test). Differences with statistical significance are shown as bold.

	**CTL DMEM**	**DMEM E_2**	**CTL HF**	**HF E_2**
CTL DMEM		**1E-20**	0.95	**1E-20**
DMEM E_2	**1E-20**		**1E-20**	**1.22E-7**
CTL HF	0.95	**1E-20**		**1E-20**
HF E_2	**1E-20**	**1.22E-7**	**1E-20**	

3.1.2. Viscoelastic properties

Table S5. Equilibrium Modulus values (in Pa) for MCF-7 cells measured with tips, shown are the number of measurements, the mean value, the standard deviation, the standard error of the mean value and the median.

	**N**	**Mean**	**SD**	**SE**	**Median**
CTL DMEM	323	572	296	16	508
DMEM E_2	352	392	200	11	348
CTL HF	339	562	298	16	509
HF E_2	335	332	161	8.8	313

Table S6. Statistical analysis of equilibrium Modulus values for MCF-7 cells (one-way ANOVA with post-hoc Tukey test). Differences with statistical significance are shown as bold.

	**CTL DMEM**	**DMEM E_2**	**CTL HF**	**HF E_2**
CTL DMEM		**1E-20**	0.95	**1E-20**
DMEM E_2	**1E-20**		**1E-20**	**0.0076**
CTL HF	0.95	**1E-20**		**1E-20**
HF E_2	**1E-20**	**0.0076**	**1E-20**	

Table S7. E_1 values (in Pa) for MCF-7 cells measured with tips, shown are the number of measurements, the mean value, the standard deviation, the standard error of the mean value and the median.

	**N**	**Mean**	**SD**	**SE**	**Median**
CTL DMEM	319	445	187	10	416
DMEM E_2	356	427	213	11	364
CTL HF	334	598	305	17	501
HF E_2	333	371	162	9	348

Table S8. Statistical analysis of E_1 values for MCF-7 cells (Kruskal-Wallis-ANOVA). Differences with statistical significance are shown as bold.

	**CTL DMEM**	**DMEM E_2**	**CTL HF**	**HF E_2**
CTL DMEM		**0.0256**	**1E-11**	**2E-7**
DMEM E_2	**0.0256**		**1.3E-17**	**0.004**
CTL HF	**1E-11**	**1.3E-17**		**1E-20**
HF E_2	**2E-7**	**0.004**	**1E-20**	

Table S9. E_2 values (in Pa) for MCF-7 cells measured with tips, shown are the number of measurements, the mean value, the standard deviation, the standard error of the mean value and the median.

	N	Mean	SD	SE	Median
CTL DMEM	316	399	175	10	375
DMEM E_2	371	380	224	12	322
CTL HF	339	510	258	14	449
HF E_2	333	304	138	8	88

Table S10. Statistical analysis of E_2 values for MCF-7 cells (Kruskal-Wallis-ANOVA). Differences with statistical significance are shown as bold.

	CTL DMEM	DMEM E_2	CTL HF	HF E_2
CTL DMEM		**0.0014**	8E-8	**3E-13**
DMEM E_2	**0.0014**		**6E-16**	**1E-4**
CTL HF	8E-8	**6E-16**		**1E-20**
HF E_2	**3E-13**	**1E-4**	**1E-20**	

Table S11. E_{inst} values (in Pa) for MCF-7 cells measured with tips, shown are the number of measurements, the mean value, the standard deviation, the standard error of the mean value and the median.

	N	Mean	SD	SE	Median
CTL DMEM	317	1403	571	32	1299
DMEM E_2	364	1236	664	35	1085
CTL HF	333	1644	764	42	1492
HF E_2	334	996	424	23	952

Table S12. Statistical analysis of E_{inst} values for MCF-7 cells (Kruskal-Wallis-ANOVA). Differences with statistical significance are shown as bold.

	CTL DMEM	DMEM E_2	CTL HF	HF E_2
CTL DMEM		**3E-7**	1.5E-4	**1E-20**
DMEM E_2	**3E-7**		**1E-16**	**3.6E-5**
CTL HF	1.5E-4	**1E-16**		**1E-20**
HF E_2	**1E-20**	**3.6E-5**	**1E-20**	

Table S13. Viscosity η_1 values (in Pa s) for MCF-7 cells measured with tips, shown are the number of measurements, the mean value, the standard deviation, the standard error of the mean value and the median.

	N	Mean	SD	SE	Median
CTL DMEM	319	81	42	2	71
DMEM E_2	373	82	51	3	69
CTL HF	340	97	56	3	81
HF E_2	335	71	36	1	65

Table S14. Statistical analysis of Viscosity η_1 values for MCF-7 cells (Kruskal-Wallis-ANOVA). Differences with statistical significance are shown as bold.

	CTL DMEM	DMEM E_2	CTL HF	HF E_2
CTL DMEM		0.40	**6.4E-4**	**0.004**
DMEM E_2	0.40		**3E-5**	0.06
CTL HF	**6.4E-4**	**3E-5**		3E-10
HF E_2	0.004	0.06	**3E-10**	

Table S15. Viscosity η_2 values (in Pa s) for MCF-7 cells measured with tips, shown are the number of measurements, the mean value, the standard deviation, the standard error of the mean value and the median.

	N	Mean	SD	SE	Median
CTL DMEM	321	1270	822	46	1068
DMEM E_2	373	1206	904	47	912
CTL HF	337	1394	834	45	1206
HF E_2	334	928	522	29	791

Table S16. Statistical analysis of Viscosity η_2 values for MCF-7 cells (Kruskal-Wallis-ANOVA). Differences with statistical significance are shown as bold.

	CTL DMEM	DMEM E_2	CTL HF	HF E_2
CTL DMEM		0.048	**0.02**	**2E-7**
DMEM E_2	0.048		**10E-6**	**0.002**
CTL HF	**0.02**	**10E-6**		**1E-15**
HF E_2	**2E-7**	**0.002**	**1E-15**	

3.2. Measurements performed with ***particles***

3.2.1. Elastic properties

Table S17. Apparent Young's Modulus values (in Pa) for MCF-7 cells measured with particles, shown are the number of measurements, the mean value, the standard deviation, the standard error of the mean value and the median.

	N	Mean	SD	SE	Median
CTL DMEM	212	309	131	9	292
DMEM E_2	385	276	145	7	240
CTL HF	260	437	206	13	407
HF E_2	345	317	137	7	296

Table S18. Statistical analysis of apparent Young's Modulus values for MCF-7 cells (Kruskal-Wallis ANOVA). Differences with statistical significance are shown as bold.

	CTL DMEM	DMEM E_2	CTL HF	HF E_2
CTL DMEM		**4.1E-4**	**1E-11**	0.62
DMEM E_2	**4.1E-4**		**1E-20**	**8E-6**
CTL HF	**1E-11**	**1E-20**		**1E-12**
HF E_2	0.62	**8E-6**	**1E-12**	

Table S19. Indentation values (in µm) for MCF-7 cells measured with particles, shown are the number of measurements, the mean value, the standard deviation, the standard error of the mean value and the median.

	N	Mean	SD	SE	Median
CTL DMEM	222	1.20	0.32	0.02	1.19
DMEM E_2	359	1.21	0.42	0.02	1.20
CTL HF	266	1.11	0.39	0.02	1.06
HF E_2	289	1.12	0.44	0.03	1.11

Table S20. Statistical analysis of apparent Young's Modulus values for MCF-7 cells (one-way ANOVA with post-hoc Tukey test). Differences with statistical significance are shown as bold.

	CTL DMEM	DMEM E_2	CTL HF	HF E_2
CTL DMEM		0.99	0.09	0.061
DMEM E_2	0.99		**0.018**	**0.0096**
CTL HF	0.09	**0.018**		0.99
HF E_2	0.061	**0.0096**	0.99	

3.2.2. Viscoelastic properties

Table S21. Equilibrium Modulus values (in Pa) for MCF-7 cells measured with particles shown are the number of measurements, the mean value, the standard deviation, the standard error of the mean value and the median.

	N	Mean	SD	SE	Median
CTL DMEM	215	99	44	3	95
DMEM E_2	396	83	51	3	68
CTL HF	252	122	73	5	108
HF E_2	360	108	58	3	89

Table S22. Statistical analysis of equilibrium Modulus values for MCF-7 cells (one-way ANOVA with post-hoc Tukey test). Differences with statistical significance are shown as bold.

	CTL DMEM	DMEM E_2	CTL HF	HF E_2
CTL DMEM		**3E-9**	**0.007**	0.60
DMEM E_2	**3E-9**		**5E-13**	**1E-11**
CTL HF	0.007	**5E-13**		**0.047**
HF E_2	0.60	**1E-11**	**0.047**	

Table S23. *E_1* values (in Pa) for MCF-7 cells measured with particles, shown are the number of measurements, the mean value, the standard deviation, the standard error of the mean value and the median.

	N	Mean	SD	SE	Median
CTL DMEM	215	86	51	3	68
DMEM E_2	396	74	45	2	57
CTL HF	252	87	44	3	76
HF E_2	360	80	38	2	70

Table S24. Statistical analysis of *E_1* values for MCF-7 cells (Kruskal-Wallis-ANOVA). Differences with statistical significance are shown as bold.

	CTL DMEM	DMEM E_2	CTL HF	HF E_2
CTL DMEM		**2E-5**	0.29	0.68
DMEM E_2	**2E-5**		**1E-6**	**2E-5**
CTL HF	0.29	**1E-6**		0.10
HF E_2	0.68	**2E-5**	0.10	

Table S25. *E_2* values (in Pa) for MCF-7 cells measured with particles, shown are the number of measurements, the mean value, the standard deviation, the standard error of the mean value and the median.

	N	Mean	SD	SE	Median
CTL DMEM	215	64	34	2	54
DMEM E_2	396	58	37	2	45
CTL HF	252	73	42	3	60
HF E_2	360	62	29	2	55

Table S26. Statistical analysis of *E_2* values for MCF-7 cells (Kruskal-Wallis-ANOVA). Differences with statistical significance are shown as bold.

	CTL DMEM	DMEM E_2	CTL HF	HF E_2
CTL DMEM		**9E-5**	**0.036**	0.79
DMEM E_2	**9E-5**		**2E-8**	**4E-5**
CTL HF	**0.036**	**2E-8**		**0.008**

HF E_2	0.79	**4E-5**	**0.008**	

Table S27. E_{inst} values (in Pa) for MCF-7 cells measured with particles, shown are the number of measurements, the mean value, the standard deviation, the standard error of the mean value and the median.

	N	Mean	SD	SE	Median
CTL DMEM	215	251	117	8	214
DMEM E_2	396	216	132	7	170
CTL HF	252	284	151	10	248
HF E_2	360	250	125	7	214

Table S28. Statistical analysis of E_{inst} values for MCF-7 cells (Kruskal-Wallis-ANOVA). Differences with statistical significance are shown as bold.

	CTL DMEM	DMEM E_2	CTL HF	HF E_2
CTL DMEM		**2E-7**	**0.036**	0.56
DMEM E_2	**2E-7**		**5E-11**	**4E-7**
CTL HF	**0.036**	**5E-11**		**0.007**
HF E_2	0.56	**4E-7**	**0.007**	

Table S29. Viscosity η_1 values (in Pa s) for MCF-7 cells measured with particles, shown are the number of measurements, the mean value, the standard deviation, the standard error of the mean value and the median.

	N	Mean	SD	SE	Median
CTL DMEM	213	13.7	6.7	0.5	12.0
DMEM E_2	394	11.9	6.5	0.3	10.2
CTL HF	253	16.2	9.6	0.6	14.1
HF E_2	359	13.4	7.3	0.4	11.8

Table S30. Statistical analysis of Viscosity η_1 values for MCF-7 cells (Kruskal-Wallis-ANOVA). Differences with statistical significance are shown as bold.

	CTL DMEM	DMEM E_2	CTL HF	HF E_2
CTL DMEM		**1.4E-4**	**0.02**	0.32
DMEM E_2	**1.4E-4**		**3E-9**	**0.002**
CTL HF	**0.02**	**3E-9**		**4.1E-4**
HF E_2	0.32	**0.002**	**4.1E-4**	

Table S31. Viscosity η_2 values (in Pa s) for MCF-7 cells measured with particles, shown are the number of measurements, the mean value, the standard deviation, the standard error of the mean value and the median.

	N	Mean	SD	SE	Median
CTL DMEM	217	230.6	162.7	11.0	180.7
DMEM E_2	393	183.9	133.2	6.7	141.2
CTL HF	252	238.6	166.3	10.5	191.6
HF E_2	359	189.4	119.1	6.3	159.9

Table S32. Statistical analysis of Viscosity η_2 values for MCF-7 cells (Kruskal-Wallis-ANOVA). Differences with statistical significance are shown as bold.

	CTL DMEM	DMEM E_2	CTL HF	HF E_2
CTL DMEM		2E-5	**0.52**	**0.0049**
DMEM E_2	2E-5		**1E-6**	**0.076**
CTL HF	**0.52**	**1E-6**		**4.1E-4**

HF E_2	**0.0049**	**0.076**	**4.1E-4**	
